# Glycine-Rich RNA-Binding Protein *AtGRP7* Functions in Nickel and Lead Tolerance in *Arabidopsis*

**DOI:** 10.3390/plants13020187

**Published:** 2024-01-10

**Authors:** Yeon-Ok Kim, Mahpara Safdar, Hunseung Kang, Jangho Kim

**Affiliations:** 1 Interdisciplinary Program in IT-Bio Convergence System, Chonnam National University, Gwangju 61186, Republic of Korea; mahpara702@gmail.com; 2 Department of Convergence Biosystems Engineering, College of Agriculture and Life Sciences, Chonnam National University, Gwangju 61186, Republic of Korea; 3 Department of Rural and Biosystems Engineering, College of Agriculture and Life Sciences, Chonnam National University, Gwangju 61186, Republic of Korea; 4 Department of Applied Biology, College of Agriculture and Life Sciences, Chonnam National University, Gwangju 61186, Republic of Korea

**Keywords:** *Arabidopsis*, *AtGRP7*, Ni, Pb, heavy metal tolerance, antioxidant enzyme

## Abstract

Plant glycine-rich RNA-binding proteins (GRPs) play crucial roles in the response to environmental stresses. However, the functions of *AtGRP7* in plants under heavy metal stress remain unclear. In the present study, in *Arabidopsis*, the transcript level of *AtGRP7* was markedly increased by Ni but was decreased by Pb. *AtGRP7*-overexpressing plants improved Ni tolerance, whereas the knockout mutant (*grp7*) was more susceptible than the wild type to Ni. In addition, *grp7* showed greatly enhanced Pb tolerance, whereas overexpression lines showed high Pb sensitivity. Ni accumulation was reduced in overexpression lines but increased in *grp7*, whereas Pb accumulation in *grp7* was lower than that in overexpression lines. Ni induced glutathione synthase genes *GS1* and *GS2* in overexpression lines, whereas Pb increased metallothionein genes *MT4a* and *MT4b* and phytochelatin synthase genes *PCS1* and *PCS2* in *grp7*. Furthermore, Ni increased *CuSOD1* and *GR1* in *grp7*, whereas Pb significantly induced *FeSOD1* and *FeSOD2* in overexpression lines. The mRNA stability of *GS2* and *PCS1* was directly regulated by AtGRP7 under Ni and Pb, respectively. Collectively, these results indicate that *AtGRP7* plays a crucial role in Ni and Pb tolerance by reducing Ni and Pb accumulation and the direct or indirect post-transcriptional regulation of genes related to heavy metal chelators and antioxidant enzymes.

## 1. Introduction

Heavy metal (HM) pollutants in the environment have increased tremendously because of industrial pollution, urban activities, and agricultural practices [1]. Soil HM contamination has become a worldwide environmental problem because HMs are not naturally degraded and accumulate continuously in the soil for a long time. Ni is an essential element for plant growth and development, is a cofactor of several enzymes, such as glyoxalase and urease, and is required in very low amounts in plants [2,3]. However, the excessive application of fertilizers and pesticides, as well as other anthropogenic activities such as battery manufacturing, has increased the release of Ni into the environment [4]. Similarly, Pb is a toxic nonessential element that is primarily released from mining, automobile exhausts, and industrial waste. Pb exerts highly toxic effects on the environment, even when introduced into plant cells in minute quantities [5]. Excess essential HMs (Ni, Mn, Zn, Fe, and Cu) and small amounts of hazardous nonessential HMs (Pb, Cd, As, and Hg) interfere with the physiological, biochemical, and metabolic processes of plants by disrupting the structural integrity of functional groups in important cellular molecules; this phenomenon inhibits important plant events, such as photosynthesis, respiration, and enzymatic activity, and negatively affects seed germination, plant growth, and production [6,7,8]. To prevent toxic reactions of HMs, plants employ complex resistance strategies, such as the chelation of cellular free HM ions via HM chelators, the sequestration of HM ions from the metabolically active cytoplasm into inactive compartments such as vacuoles and cell walls, and the homeostasis of HM-generated reactive oxygen species (ROS) [8,9,10].

HM chelation is a powerful strategy for reducing free toxic HM ions in plant cells. Glutathione (GSH), phytochelatins (PCs), and metallothioneins (MTs) are HM-binding ligands that protect plant cells from the toxicity of free HM ions by complexing with and sequestering toxic metals [11,12]. The expression levels of genes associated with the syntheses of GSH (GSH synthetase, GS), PC (PC synthase, PCS), and MT in plants are usually dynamic in response to HM stress. These genes play pivotal roles in the detoxification and tolerance of HMs [11,12,13,14]. Moreover, high HM concentrations in plant cells induce oxidative stress by promoting ROS generation, which can damage cellular macromolecules, including lipids, proteins, and DNA, and cause the denaturation of cell structures and membranes, leading to cell death and plant growth inhibition [15]. Therefore, ROS removal is another strategy to alleviate HM toxicity. ROS scavenging is accompanied by an antioxidant system composed of enzymatic and nonenzymatic antioxidants that maintain the redox state of the cell. Antioxidant enzymes, including superoxide dismutase (SOD), which dismutates O_2_^−^ into H_2_O_2_, and peroxidase (POD), glutathione reductase (GR), and catalase (CAT), which convert H_2_O_2_ into H_2_O and O_2_, alleviate the adverse effects of oxidative stress and stabilize cell membranes in plants under HM stress [16,17,18]. Plant responses and adaptations to environmental stresses result from alterations in gene expression via transcriptional and post-transcriptional regulation in plants [19,20,21,22]. Therefore, understanding the expression regulation of genes associated with HM chelators and antioxidant enzymes is important to understand the adaptation and tolerance mechanisms of plants under Ni and Pb stress.

Glycine-rich RNA-binding proteins (GRPs), which contain an RNA-binding domain at the N-terminus and a glycine-rich region at the C-terminus, play important roles in RNA metabolism at the transcriptional and post-transcriptional levels via either the direct or indirect binding of target RNAs under environmental stresses, such as cold temperatures, drought, wounding, UV radiation, and high salinity [23,24,25,26,27,28]. GRPs are involved in abiotic stress tolerance, but their functional roles in response to HMs are unclear. The expression levels of two maize GRPs are downregulated by Cr stress [29], whereas the expression of soybean GRP-like gene (*GmGRPL*) is upregulated by Al stress [30]. In addition, *AtGRP3* knockdown and *GmGRPL* overexpression enhance Al tolerance in *Arabidopsis* [30,31]. The expression of *AtGRP7*, a well-characterized GRP in *Arabidopsis*, is altered by various stresses, such as cold, high salinity, or drought, and affects the stress tolerance in *Arabidopsis* [32] by RNA chaperone activity that regulates the export of mRNA. However, to date, the role of *AtGRP7* in HM stress response remains to be elucidated.

Hence, the present study aimed to explore the functional roles of *AtGRBP7* under Ni and Pb stress. We found that *AtGRP7*-overexpressing *Arabidopsis* showed enhanced Ni tolerance, whereas the *grp7* knockout mutant was more susceptible than the wild type to Ni stress. The *grp7* mutant showed higher tolerance to Pb than to Ni, whereas the overexpressing plants showed Pb sensitivity. To elucidate the molecular mechanisms underlying *AtGRP7*-mediated Ni and Pb tolerance, the expression modulation of genes related to HM chelators and antioxidant enzymes was analyzed in the *grp7* mutant and overexpressing lines during Ni and Pb exposure.

## 2. Results

### 2.1. Effects of Ni and Pb Stress on the Transcript Level of AtGRP7

**Figure 1:**
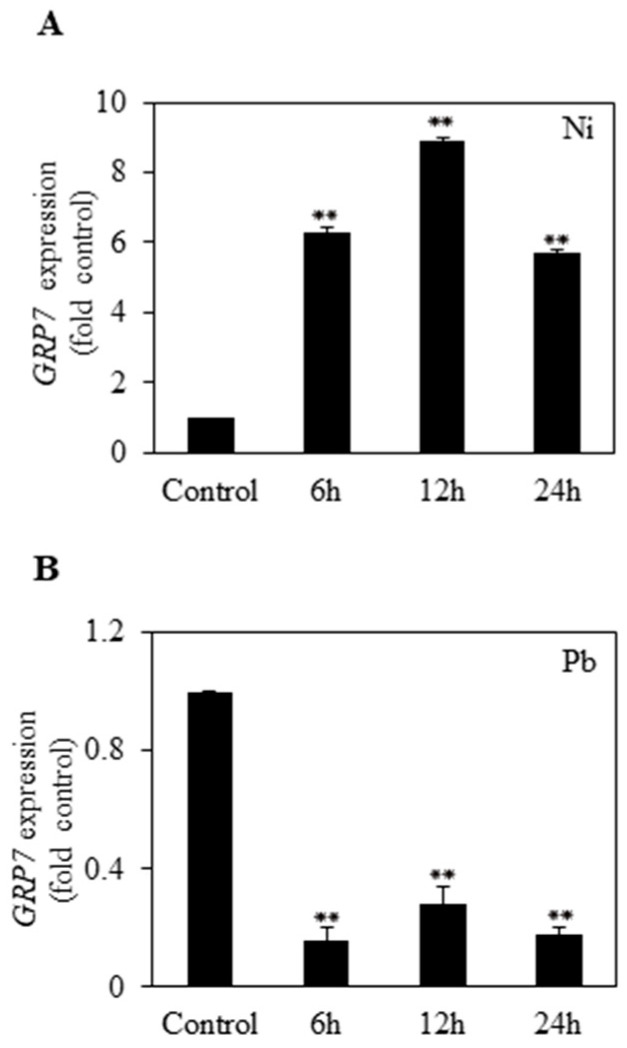
Expression level of *AtGRP7* under Ni and Pb stress. Fourteen-day-old *Arabidopsis* seedlings were treated with 100 μM Ni (**A**) and 500 μM Pb (**B**) for 6 h, 12 h, and 24 h, and transcript level of *AtGRP7* was determined via qRT-PCR. Each value is mean ± SD of three replicates, and asterisks indicate significant differences compared with control (*p* < 0.01).

To determine the effects of Ni and Pb treatments on the expression pattern of *AtGRP7* in *Arabidopsis*, we treated 14-day-old *Arabidopsis* seedlings with 100 μM Ni and 500 μM Pb for different time periods (6, 12, and 24 h). qRT-PCR analysis showed that the transcript level of *AtGRP7* was quickly upregulated by approximately 6-fold after 6 h of Ni treatment and further increased by approximately 9-fold at 12 h. After 24 h of Ni treatment, the expression of *AtGRP7* gradually decreased by 6-fold (Figure 1A). By contrast, Pb treatment greatly reduced the transcript level of *AtGRP7* within 6 h, and this decrease was maintained until 24 h (Figure 1B).

### 2.2. Effects of AtGRP7 on Germination and Seedling Growth under Ni and Pb Stress

**Figure 2:**
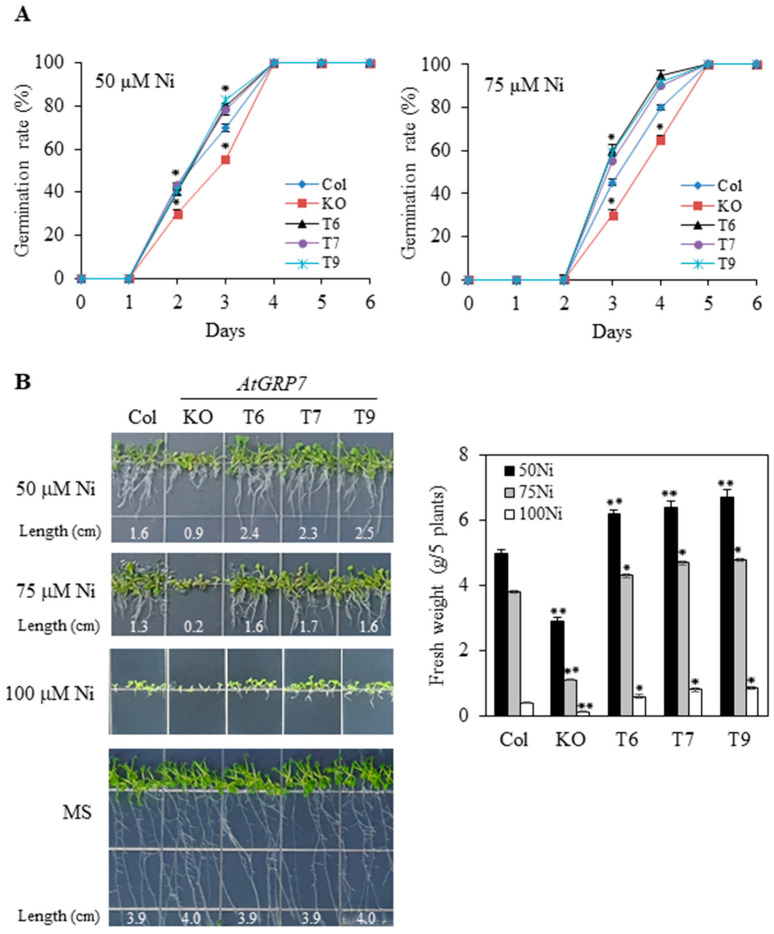
Effects of Ni stress on seed germination and seedling growth of wild type, mutant, and transgenic plants. (**A**) Wild type (Col), *grp7* mutant (KO), and *AtGRP7*-overexpressing lines (T6, T7, and T9) were sown on 1/2 MS medium containing 50 or 75 μM NiCl_2_, and seed germination rate was scored. (**B**) The germinated seeds were grown vertically, and primary root length and fresh weight of the seedlings were measured on day 10 of transplantation. Each value is mean ± SD of three replications, and asterisks indicate significant differences compared with wild type (* *p* < 0.05, ** *p* < 0.01).

To elucidate the functional role of *AtGRP7* in response to Ni and Pb stress, we used three *AtGRP7*-overexpressing lines (T6, T7, and T9) and a *grp7* knockout mutant as described in a previous study [32]. All genotypes showed similar germination rates and seedling growth under control conditions. Low concentrations of Ni (~20 μM) did not affect the germination rate or seedling growth in the wild type, *grp7* mutant, or overexpression lines. Under 50 and 75 μM Ni, the germination rate of the *AtGRP7*-overexpressing lines was better, but the *grp7* mutant showed slightly inhibited seed germination compared to the wild type (Figure 2A). In comparison with 50 and 75 μM Ni, 100 μM Ni remarkably retarded the germination of all genotypes and did not show significant differences in each genotype. Under 50, 75, or 100 μM Ni, the biomass including seedling growth and fresh weight of the *AtGRP7*-overexpressing lines was higher than that of the wild type. However, compared with that shown by the wild type, the *grp7* mutant showed markedly retarded seedling growth and fresh weight (Figure 2B).

**Figure 3:**
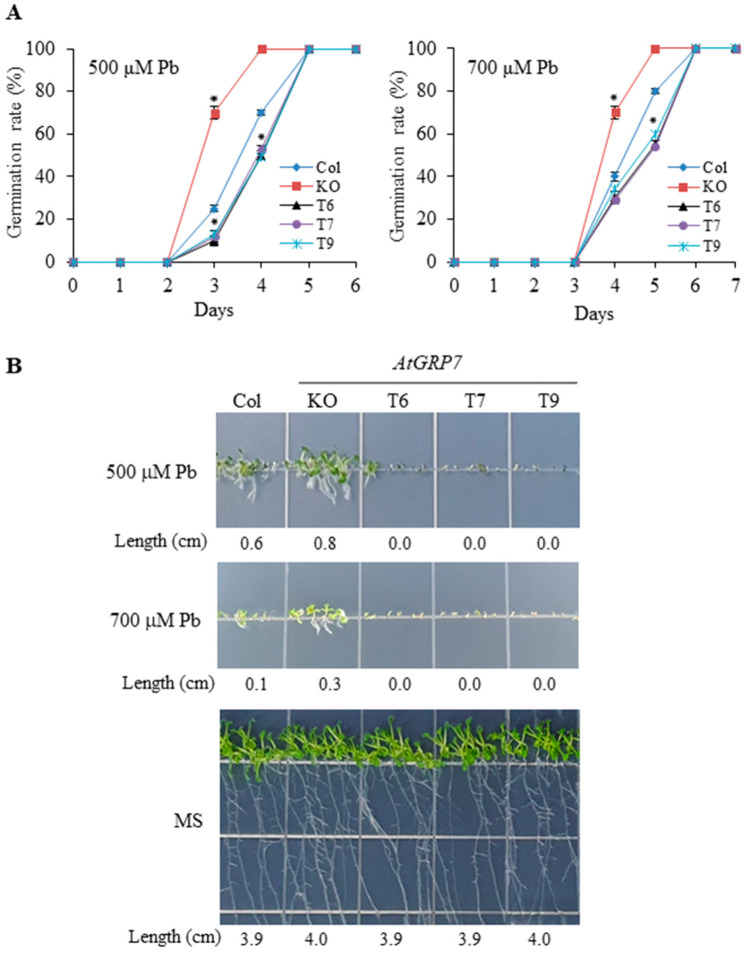
Effects of Pb stress on seed germination and seedling growth of wild type, mutant, and transgenic plants. (**A**) Wild type (Col), *grp7* mutant (KO), and *AtGRP7*-overexpressing lines (T6, T7, and T9) were sown on 1/2 MS medium containing 500 or 700 μM PbSO_4_ and seed germination rate was scored. Each value is mean ± SD of three replications, and asterisks indicate significant differences compared with wild type (*p* < 0.05). (**B**) The germinated seeds were grown vertically, and primary root lengths of the seedlings were measured on day 10 of transplantation.

When the three genotypes were grown on solid medium supplemented with 500 or 700 μM Pb, the germination of the *grp7* mutant was strongly promoted, but that of the *AtGRP7*-overexpressing lines was more delayed compared with that of the wild type (Figure 3A). In particular, Pb stress remarkably suppressed the post-germination seedling growth of the *AtGRP7*-overexpressing lines but considerably improved that of the *grp7* mutant (Figure 3B). Notably, under 700 μM Pb, the cotyledons of the *grp7* mutant grew well with cotyledon greening, whereas those of the overexpression lines did not show post-germination development.

### 2.3. Bioaccumulation of Ni and Pb

**Figure 4:**
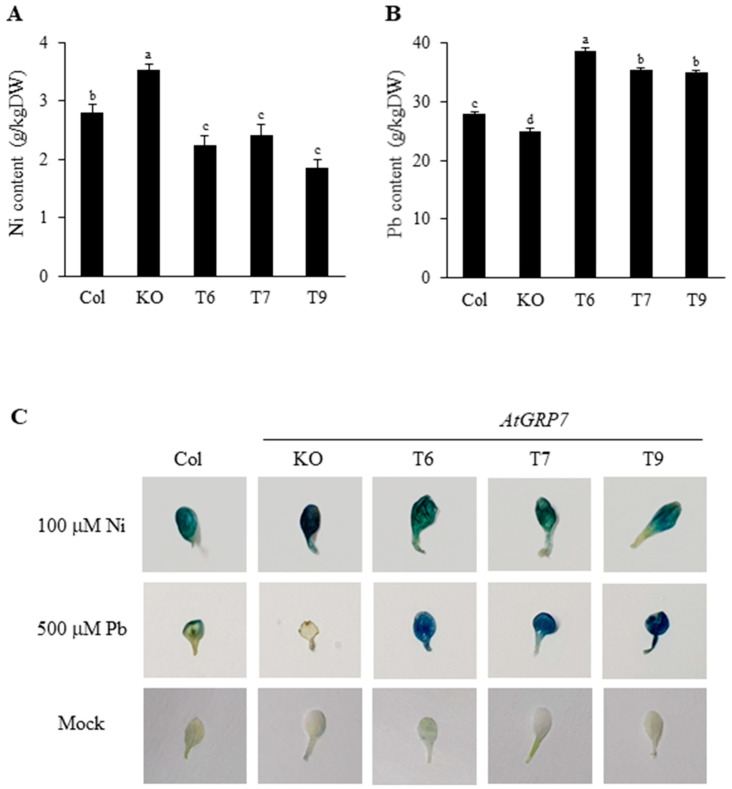
Ni and Pb accumulation and cell damage in wild type, mutant, and transgenic plants. Fourteen-day-old seedlings of wild type (Col), *grp7* mutant (KO), and *AtGRP7*-overexpressing lines (T6, T7, and T9) were treated with 100 μM Ni (**A**) and 500 μM Pb (**B**) for 24 h. Each value is mean ± SD of three independent experiments, and different letters indicate significant differences between different genotypes (*p* < 0.05, Duncan’s multiple range test). (**C**) Visualization of cell death in leaves of *Arabidopsis* via Evans blue staining.

To determine the effect of *AtGRP7* on Ni and Pb uptake, we treated the seedlings of the three genotypes with 100 μM Ni and 500 μM Pb for 24 h (Figure 4A,B). Ni accumulation increased in the *grp7* mutant but decreased in the *AtGRP7*-overexpressing lines compared with that in the wild type (Figure 4A). The *AtGRP7*-overexpressing lines accumulated more Pb than Ni, whereas the *grp7* mutant accumulated less Pb than Ni when compared with those in the wild type (Figure 4B).

High concentrations of Ni and Pb in plant cells affect the cellular metabolism and cause cellular damage. To determine whether Ni and Pb can cause cell damage, we treated the seedlings of the three genotypes with 100 μM Ni and 500 μM Pb for 24 h and then stained them with Evans blue, a dye that stains dead cells blue (Figure 4C). The Ni-treated *grp7* mutant showed a significantly stronger density of staining in the leaves than that of the Ni-treated wild type, whereas the *AtGRP7*-overexpressing lines exhibited a lower density of staining in the leaves. Under Pb treatment, the leaves of the *AtGRP7*-overexpressing lines showed strong staining density, but the *grp7* mutant displayed a much lower staining density than that of the wild type (Figure 4C).

### 2.4. Effects of AtGRP7 on Transcript Levels of HM Chelators under Ni and Pb Stress

**Figure 5:**
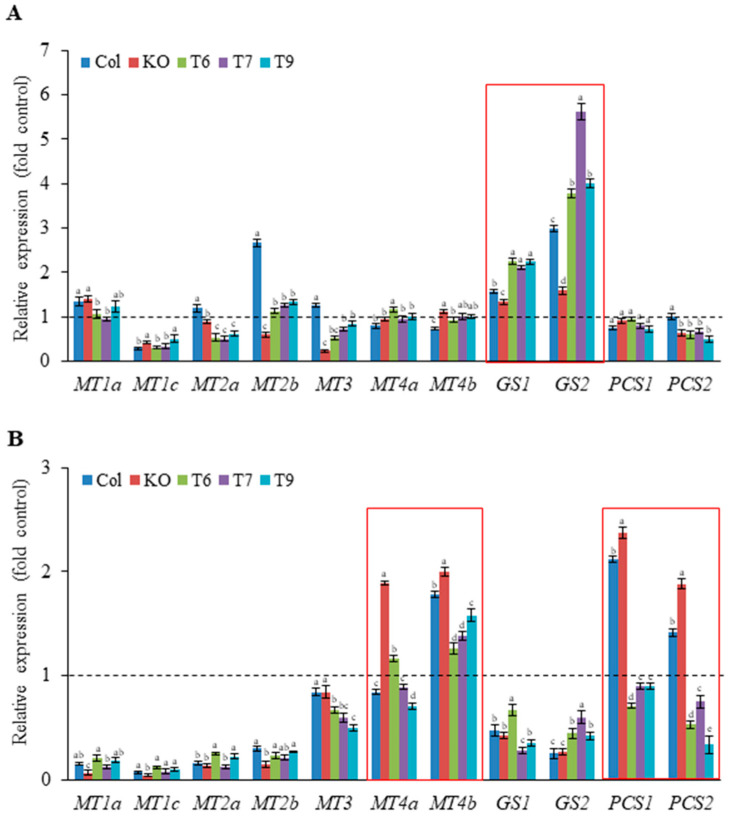
Effects of Ni and Pb on the expression levels of heavy metal chelators in wild type, mutant, and transgenic plants. Fourteen-day-old seedlings of wild type (Col), *grp7* mutant (KO), and *AtGRP7*-overexpressing lines (T6, T7, and T9) were treated with 100 μM Ni (**A**) and 500 μM Pb (**B**) for 24 h. The transcript levels of MT, GSH, and PC synthesis genes were analyzed via qRT-PCR and were normalized to mRNA level of actin, used as internal control. Mock-treated wild type was set to 1 as a control for each gene (dotted line). Each value is the mean ± SD of three replicates and different letters indicate significant differences between different genotypes (*p* < 0.05, Duncan’s multiple range test).

HM chelation reduces the intracellular toxicity of free Ni and Pb ions and increases the tolerance of plants to these HMs. To explore the function of *AtGRP7* in Ni and Pb tolerance, we measured the transcript levels of genes related to major HM chelators, such as GSH, PCs, and MT, through qRT-PCR (Figure 5). Under control conditions, *AtGRP7* downregulated *MT1c* and *MT2a*, whereas it upregulated *MT2b* in the *grp7* mutant and *MT4b* in *AtGRP7*-overexpressing lines (Figure S1). Under Ni stress, none of the genotypes displayed significant alterations in *MT1a*, *MT4a*, or *MT4b* expression. However, all genotypes showed a reduction in *MT1C* expression (Figure 5A). Meanwhile, Ni stress significantly reduced the expression of *MT2a* in the *AtGRP7*-overexpressing lines and the expression of *MT3* in the mutant and *AtGRP7*-overexpressing lines. Although the *MT2b* expression of the *AtGRP7*-overexpressing lines did not change, the *MT2b* expression of the wild type increased by 2.7-fold, whereas that of the *grp7* mutant decreased. Interestingly, the *GS1* and *GS2* expression in the *AtGRP7*-overexpressing lines greatly increased by approximately 2-fold and 4–6-fold, respectively. The wild type also showed 1.6- and 3-fold upregulation in *GS1* and *GS2* expression, respectively. However, the transcripts in the *grp7* mutant did not show any changes (Figure 5A). By contrast, the expression levels of the PC synthesis genes (*PCS1* and *PCS2*) were slightly reduced or remained unchanged in all genotypes. Under Pb stress, the expression levels of *MT1a*, *MT1c*, *MT2a*, and *MT2b* were severely downregulated in all genotypes, whereas the expression of *MT3* was only slightly reduced in the *AtGRP7*-overexpressing lines (Figure 5B). The expression levels of *MT4a* and *MT4b* were increased by 2-fold in the *grp7* mutant, which were higher than those in the wild type and *AtGRP7*-overexpressing lines. The expression levels of *GS1* and *GS2* were significantly downregulated in all genotypes. Pb stress increased *PCS1* expression by approximately 2-fold in the wild type and the mutant and *PCS2* expression in the mutant. However, it reduced the expression levels of *PCS1* and *PCS2* in the *AtGRP7*-overexpressing lines, which were lower in the mutant (Figure 5B).

### 2.5. Effects of AtGRP7 on Transcript Levels of Antioxidant Enzymes under Ni and Pb Stress

**Figure 6:**
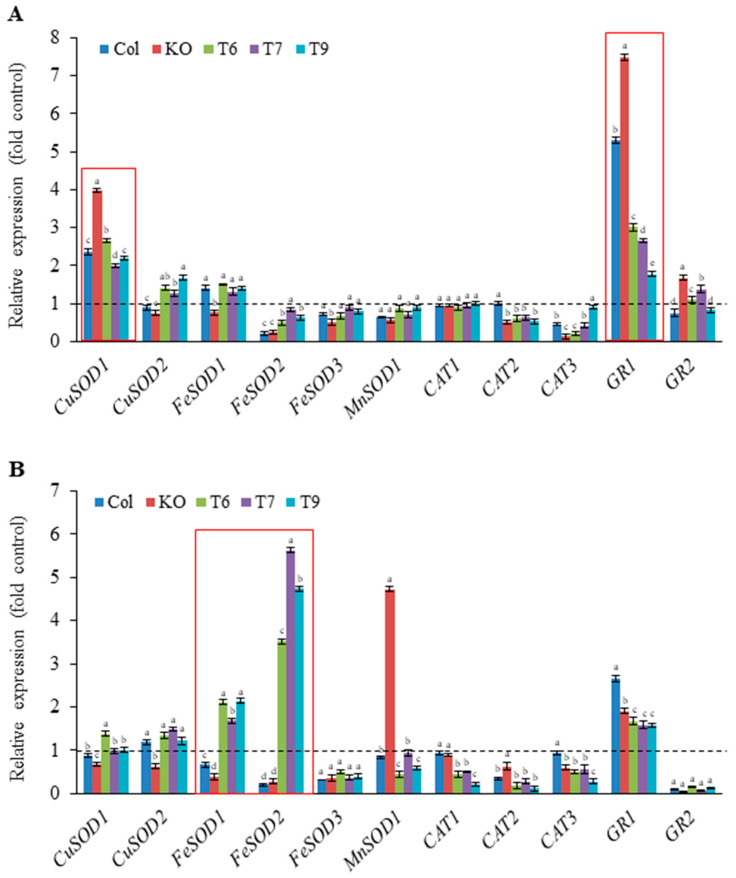
Effects of Ni and Pb on the expression levels of antioxidant enzymes in wild type, mutant, and transgenic plants. Fourteen-day-old seedlings of wild type (Col), *grp7* mutant (KO), and *AtGRP7*-overexpressing lines (T6, T7, and T9) were treated with 100 μM Ni (**A**) and 500 μM Pb (**B**) for 24 h. Expression levels of the enzymes were normalized to mRNA level of actin, used as internal control. Mock-treated wild type was set to 1 as a control for each gene (dotted line). Each value is the mean ± SD of three replicates, and different letters indicate significant differences between different genotypes (*p* < 0.05, Duncan’s multiple range test).

Excess Ni and Pb cause oxidative cellular damage through ROS generation; thus, understanding the transcriptional regulation of antioxidant enzyme genes involved in ROS scavenging is important. Under control conditions, *AtGRP7* downregulated *FeSOD1* and *FeSOD2* in the *grp7* mutant, whereas it upregulated *CAT3* in *AtGRP7*-overexpressing lines (Figure S1). The transcript levels of *SODs*, *CATs*, and *GRs* in the Ni- and Pb-treated seedlings are shown in Figure 6. Ni stress increased *CuSOD1* expression by approximately 2-fold in the wild type and *AtGRP7*-overexpressing lines and by 4-fold in the *grp7* mutant (Figure 6A). However, it downregulated the expression of *FeSOD2*, *FeSOD3*, *MnSOD1*, and *CAT3* and did not change the expression of *CAT1* in all genotypes. Meanwhile, under the same treatment, the *CuSOD2* and *FeSOD1* expression levels were only slightly higher in the *AtGRP7*-overexpressing lines than in the *grp7* mutant. *CAT2* expression did not change in the wild type but was reduced in the *grp7* mutant and *AtGRP7*-overexpressing lines. Notably, *GR1* expression increased in the wild type and *grp7* mutant by approximately 5.3-fold and 7.5-fold, respectively, but only by 1.5–3.0-fold in the *AtGRP7*-overexpressing lines (Figure 6A). *GR2* expression only slightly increased in the mutant. Under Pb stress, *CuSOD1* and *CuSOD2* expression did not show significant alterations in the wild type and *AtGRP7*-overexpressing lines but decreased in the mutant. *FeSOD1* and *FeSOD2* expression significantly decreased in the wild type and *grp7* mutant but increased in the *AtGRP7*-overexpressing lines by 2- and 3.5–5.6-fold, respectively. Moreover, Pb stress significantly reduced *FeSOD3* and *CAT2* expression in all genotypes. *CAT1* and *CAT3* expression decreased in the *AtGRP7*-overexpressing lines and *grp7* mutant but did not change in the wild type. Interestingly, *MnSOD1* expression increased by approximately 5-fold in the *grp7* mutant but decreased in the wild type and *grp7* mutant. Furthermore, *GR1* expression increased in the wild type (2.7-fold), *grp7* mutant (1.9-fold), and *AtGRP7*-overexpressing lines (1.6–1.7-fold), whereas *GR2* expression markedly decreased in all the three genotypes (Figure 6B).

### 2.6. Effects of AtGRP7 on mRNA Stability under Ni and Pb Stress

**Figure 7:**
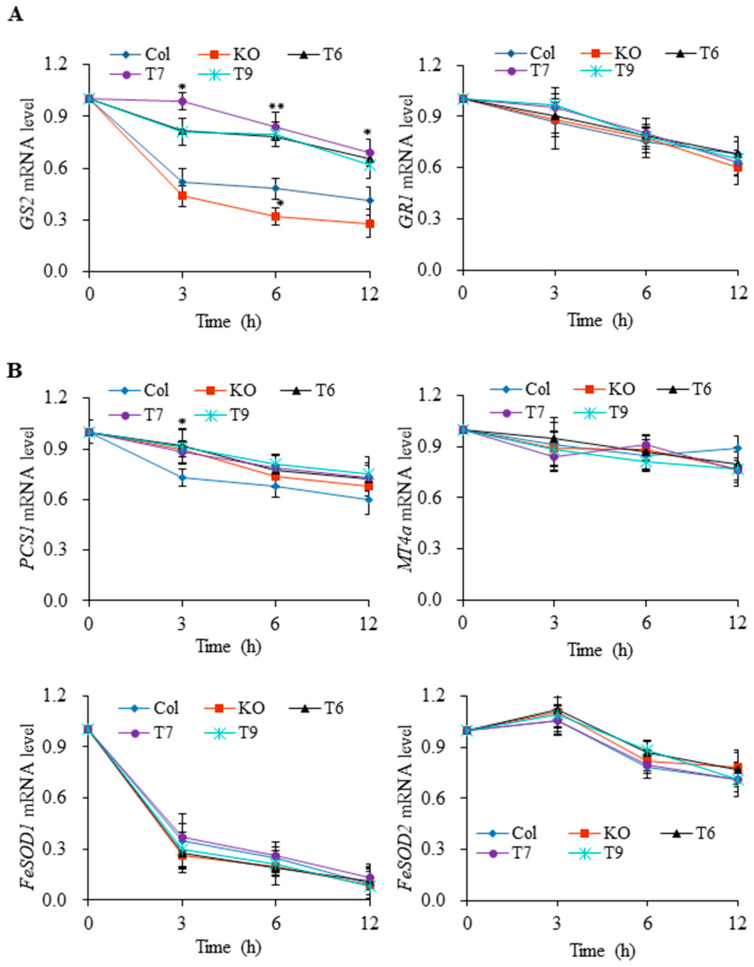
mRNA stability of target genes via AtGRP7. Two-week-old seedlings of wild type (Col), *grp7* mutant (KO), and *AtGRP7*-overexpressing lines (T6, T7, and T9) were subjected to 100 μM Ni (**A**) and 500 μM Pb (**B**) with transcription inhibitors, and the target mRNA levels were examined at different time points. Each value is mean ± SD of three replicates, and asterisks indicate significant differences compared with control (* *p* < 0.05, ** *p* < 0.01).

To understand the molecular mechanism of AtGRP7 on the post-transcriptional regulation, we examined whether AtGRP7 affects the stabilization of mRNAs such as *GS2, GR1, MT4a*, *PCS1*, *FeSOD1*, and *FeSOD2* which shows large alteration under Ni or Pb stress (Figure 7). Transcription inhibitors (TI) including actinomycin D and cordycepin effectively suppressed the transcript levels of all genes in all genotypes. Similarly, the application of Ni or Pb with TI gradually decreased the transcript levels of all target genes in all genotypes (Figure 7). *GS2* transcripts were more stable in the *AtGRP7*-overexpressing lines but degraded more rapidly in the mutant compared to the wild type in the presence of TI and Ni (Figure 7A). These results suggest that the *GS2* transcript is directly regulated by an AtGRP7-mediated mRNA stabilization. We found that the *PCS1* transcript was more reduced in the wild type than the mutant and *AtGRP7*-overexpressing lines at 3 h. However, no noticeable differences were observed between the wild type, mutant, and *AtGRP7*-overexpressing lines in mRNA stability of *GR1* under Ni stress as well as *MT4a*, *FeSOD1*, and *FeSOD2* under Pb stress. Among the genes, stability of *FeSOD1* decreased much more rapidly under Pb stress (Figure 7B).

## 3. Discussion

Although previous studies have revealed the important role of *AtGRP7* in the response to abiotic stress [32], the functional role of *AtGRP7* under HM stress has not been explored in plants. This study is the first to demonstrate a tolerance mechanism for Ni and Pb stress governed by *AtGRP7* in *Arabidopsis*. In the present study, the transcript levels of *AtGRP7* were markedly upregulated by Ni but downregulated by Pb (Figure 1). It is likely that several *cis*-regulatory elements, such as DPBF1&2, MYB4, RAV1-A, bHLH, GATA and W-box, in the promoter of *AtGRP7* are associated with the differential regulation of *AtGRP7* in response to Ni and Pb stress. It would be interesting to further explore which *cis*-regulatory elements are responsible for the altered expression patterns of *AtGRP7* in response to Ni and Pb stress. The different regulation of *AtGRP7* by Ni and Pb may lead to contrasting functions of *AtGRP7* in Ni and Pb responses, as revealed by phenotype analysis. *AtGRP7* overexpression enhanced germination and seedling growth under Ni stress but had detrimental effects on these processes under Pb stress. In contrast, the seedling growth of the *grp7* mutant was severely inhibited by Ni stress but promoted by Pb stress (Figure 2 and Figure 3). These findings suggest that *AtGRP7* functions as a positive and negative regulator of Ni and Pb tolerance, respectively. As *AtGRP7* plays an important role under Ni and Pb stress, the response mechanisms of the *AtGRP7*-overexpressing lines and *grp7* mutant need to be elucidated.

High concentrations of intracellular Ni and Pb affect various metabolic pathways, because of their strong affinity for the –SH functional groups of essential biomolecules, negatively affecting biological metabolism and plant growth [14,33]. Therefore, restricting the entry of Ni and Pb is important for Ni and Pb stress tolerance in plants. Our findings showed a correlation between maintenance of low Ni/Pb content and Ni/Pb tolerance. A low Ni accumulation in the *AtGRP7*-overexpressing lines and high Ni contents in the *grp7* mutant indicate that AtGRP7 acts as a negative regulator of Ni uptake. By contrast, a high Pb accumulation in the *AtGRP7*-overexpressing lines and low Pb contents in the *grp7* mutant indicates that AtGRP7 acts as a positive regulator of Pb uptake (Figure 4A,B). These opposite accumulation patterns of Ni and Pb result in contrasting cell damage in the *AtGRP7*-overexpressing lines and *grp7* mutant under Ni and Pb stress (Figure 4C). It is likely that AtGRP7 directly or indirectly regulates Ni and Pb uptake-related genes such as transporters. Further exploration of how AtGRP7 regulates the expression of heavy metal transporters would be an interesting future experiment.

In general, plants produce low-molecular-weight thiols, such as GSH, PCs, and MT, which have a high affinity for HMs to reduce the toxicity of free HM ions in plant cells, conferring HM tolerance to plants [11,14]. Ni-elevated GSH synthesis enhances Ni tolerance in some plants, such as mustard, *Arabidopsis*, and *Thlaspi goesingense* [34,35]. Our results showed that Ni significantly increased the expression levels of GSH synthesis genes, especially *GS2*, without affecting the upregulation of MT and PC synthesis genes in the *AtGRP7*-overexpressing lines (Figure 5A). These results suggest that GSH is important for Ni responseas reported in previous studies, and that GSH is preferentially used over MTs and PCs to reduce Ni toxicity and improve Ni tolerance. Interestingly, unlike Ni, Pb stimulated the MT and PC synthesis pathways more than the GSH synthesis pathway in the grp7 mutant (Pb tolerant). Although *MT4a* and *MT4b* were significantly induced by Pb in *grp7* mutant, the expression level of *MT4b* in the *AtGRP7*-overexpressing lines remained 2-fold higher than that of the *grp7* mutant under control conditions (Figure 5B and Figure S1). It can be interpreted that *MT4b* induction via Pb in the *grp7* mutant is much greater than the results shown in Figure 5B. Additionally, PC synthases (*PCS1* and *PCS2*) in the *grp7* mutant were significantly higher than those in the *AtGRP7*-overexpressing lines. These results suggest that MT and PC are more crucial than GSH in Pb chelation, possibly reducing the deleterious effects of Pb toxicity such as cell damage and improving the Pb tolerance of the grp7 mutant.

Ni and Pb induce ROS generation in plant cells and induce oxidative stress, which damages proteins, lipids, membranes, and nucleic acids, leading to cell death [15,33,36]. The dark color after Evans blue staining of the leaves from the Ni-treated *grp7* mutant and Pb-treated *AtGRP7*-overexpressing lines suggested an abundance of oxidative stress and severe cell damage (Figure 4C), whereas a relatively light color of staining was observed in the leaves of the Ni-treated *AtGRP7*-overexpressing lines and Pb-treated *grp7* mutant. This result suggests that Ni and Pb tolerance is correlated with reduced oxidative damage, which is consistent with the results of previous studies [37,38]. Given that antioxidant enzymes can scavenge ROS to mitigate cellular damage, whether Ni and Pb tolerance is related to the gene expression of ROS-scavenging enzymes is worth investigating. Ni and Pb stimulate the activities and gene expression of antioxidant enzymes [3,36]. Thus, the active enzymatic ROS scavenging system provides a protective response to Ni and Pb stress and confers Ni and Pb tolerance. In this study, Ni decreased the expression of most genes (*CuSOD2*, *FeSOD2*, *FeSOD3*, *MnSOD1*, and *CAT3*) in the wild type, while it significantly upregulated the expression of CuSOD1 and GR1 (Figure 6A). Inconsistent with the findings from the present study, a previous study reported that Ni upregulates *CAT, POD, APX*, and *GR* but downregulates *SOD* in rice leaves [39]. The present and previous results suggest that antioxidant enzyme genes are differentially regulated by Ni depending on the plant species and that *GR* is a crucial Ni-inducible gene in both rice and *Arabidopsis*. Interestingly, *CuSOD1* and *GR1* induced by Ni in the wild type were also significantly increased by Ni to a much higher level in the *grp7* mutant compared to the *AtGRP7*-overexpressing lines. On the other hand, the *AtGRP7*-overexpressing lines showed considerably higher *FeSOD1* and *FeSOD2* expression than the *grp7* mutant under Pb stress (Figure 6B). These results suggest that Ni- and Pb-sensitive plants may more likely suffer from the overproduction of ROS, such as O_2_^−^ and H_2_O_2_, than Ni- and Pb-tolerant plants and that, presumably, the induced ROS-scavenging genes in Ni- and Pb-sensitive plants can be utilized to overcome excessive oxidative stress induced by Ni and Pb. By contrast, the significant induction of *MnSOD1* may play a partial role in ROS scavenging in the *grp7* mutant. *AtGRP7*-mediated antioxidant enzyme gene expression shows various patterns depending on the genotype; hence, the correlation between antioxidant enzyme gene regulation and Ni or Pb tolerance is difficult to explain.

Post-transcriptional RNA regulation, including pre-mRNA splicing, polyadenylation, mRNA transport, and stability, is a fundamental step in gene expression regulation. Since it is believed that GRPs are involved in post-transcriptional RNA regulation [27], we examined whether AtGRP7 contributes to the mRNA stability of target gene transcripts which shows strong modulation during Ni and Pb stress conditions. It is obvious that the *GS2* transcript was decreased more rapidly in the *grp7* mutant than *AtGRP7*-overexpressing lines under Ni stress (Figure 7), implying that AtGRP7 directly contributes to the mRNA stability of *GS2*, resulting in an increase in *GS2* transcripts (Figure 5). More stable *PCS1* in the mutant than in the wild type over 3 h under Pb stress is believed to contribute to maintaining the high expression level of *PCS1* (Figure 5 and Figure 7). However, no significant differences in target mRNA decay among the wild type, mutant, and *AtGRP7*-overexpressing lines were found in most genes such as *GR1* under Ni stress as well as *MT4a*, *FeSOD1*, and *FeSOD2* under Pb stress, suggesting that AtGRP7 does not influence the stability of the target mRNAs but may affect other RNA regulation mechanisms such as splicing. Taken together, these results indicate that AtGRP7 differently regulates the stability of target mRNAs. To better understand the post-transcriptional RNA regulation of target genes by AtGRP7, questions of whether AtGRP7 can directly bind to these RNAs and how AtGRP7 affects the stability or splicing of its target transcripts under Ni and Pb stress warrant further studies. In addition, it would be interesting to further explore whether AtGRP7 can affect the protein level of heavy metal chelators and the activity of antioxidant enzymes involved in Ni and Pb response.

## 4. Materials and Methods 

### 4.1. Plant Materials and Growth Conditions

*Arabidopsis thaliana* Columbia-0 (Col-0) ecotype was used in this study. *Arabidopsis AtGRP7* T-DNA-tagged knockout mutant (KO, PST18261) and *AtGRP7*-overexpressing transgenic plants (T6, T7, and T9) were used throughout this study, as described previously [32]. The seeds were sterilized using 70% ethanol and 1% NaClO and then washed with sterile water several times. The seeds were sown on half-strength Murashige and Skoog (MS) medium containing 3% sucrose and were stratified at 4 °C for 3 days in darkness. The seeds were grown in a growth room maintained at 22 °C under long day conditions (16 h light/8 h dark photoperiod cycle).

### 4.2. Germination and Seedling Growth Assays under Ni and Pb Stress

Wild type, mutant, and transgenic seeds were harvested at the same time and were used for the analysis of germination and seedling growth. To determine the effect of Ni and Pb stress on germination and seedling growth, the sterilized seeds were sown on a half-strength MS agar medium supplemented with various concentrations of NiCl_2_ (50, 75, and 100 μM) and PbSO_4_ (500 and 700 μM). The seeds were stratified at 4 °C for 3 days and transferred to 23 °C under long-day conditions (16 h light/8 h dark photoperiod cycle). The germination rate was investigated for 6 days. For phenotypic analysis of post-germination growth, the germinated seeds were vertically grown, and then the root length and fresh weight of the seedlings were measured. At least three independent replicates were performed for all experiments.

### 4.3. Ni and Pb Treatment

To examine the heavy metal content and gene expression, wild type, mutant, and transgenic seeds were placed on the filter papers supplemented with MS agar medium and allowed to germinate and grow. Fourteen-day-old plants on the filter papers were transferred and treated with solutions containing 100 µM Ni or 500 µM Pb for 24 h. The harvested samples were used for RNA isolation and ICP analysis.

### 4.4. Determination of Ni and Pb Accumulation 

Ni or Pb-treated plants were rinsed several times with deionized water and dried for 72 h in an oven at 65 °C. The dried samples were milled to powder, and Ni and Pb concentrations were detected via inductively coupled plasma-spectrometry (ICP, Perkin Elmer Optima 2200 DV, Waltham, MA, USA). The entire experiment was replicated three times.

### 4.5. Evans Blue Staining Analysis

The cell viabilities of the plant leaves were determined using the Evans blue staining method [40]. Seedlings with similar growth were treated with 100 μM Ni and 500 μM Pb for 24 h, and the seedlings were stained with 0.25% (*w*/*v*) Evans blue dye for 10 min. The surface of the stained samples was washed three times with water and then observed.

### 4.6. Gene Expression Analysis

Total RNA was extracted from the harvested *Arabidopsis* seedlings using the RNeasy Plant Mini Kit (Qiagen, Valencia, CA, USA) according to the manufacturer’s instructions. Real-time PCR analysis was performed using the 2X Real-Time PCR smart mix (SolGent, Daejeon, Republic of Korea) and conducted using the Rotor-Gene Q PCR cycler (Qiagen). Actin was used as the internal control. The primers used for the qRT-PCR analysis are listed in Supplementary Table S1. Three independent replicates were performed for all experiments with different RNA samples.

### 4.7. mRNA Stability Analysis

Fourteen-day-old seedlings of wild type, mutant, and *AtGRP7*-overexpressing lines growing on half-strength MS medium were transferred to liquid half-strength MS medium for 1 h and harvested as time 0 h controls. Subsequently, transcription inhibitors (100 µM cordycepin and 10 µM actinomycin D) used to block the transcription of genes were added to the liquid medium containing 100 µM Ni or 500 µM Pb. The total RNAs extracted from the harvested samples at 0 h, 3 h, 6 h, and 12 h were used for qRT-PCR assay to elucidate the stability of target gene transcripts.

### 4.8. Statistical Analysis

All experiments were conducted with at least three repetitions. All data are presented as the means ± standard deviations. Statistically significant differences among treatments were analyzed using ANOVA (SPSS for Windows version 19) followed by Student’s *t*-test analysis (SigmaPlot version 13 (Systat Software Inc., San Jose, CA, USA) and defined as *p* < 0.05 in Figure 4, Figure 5 and Figure 6.

## Data Availability

Data are contained within the article and Supplementary Materials.

## Supplementary Materials

The following supporting information can be downloaded at: https://www.mdpi.com/article/10.3390/plants13020187/s1, Table S1: Primers for qRT-PCR used in this study; Figure S1: Expression levels of heavy metal chelators (A) and antioxidant enzymes (B) under normal conditions. Each value is the mean ± SD of three replicates.
