# Supplementary material for: Glycine-Rich RNA-Binding Protein AtGRP7 Functions in Nickel and Lead Tolerance in Arabidopsis
Source: Plants (Basel). 2024 Jan 10;13(2):187. doi: 10.3390/plants13020187 (PMC10818801; doi:10.3390/plants13020187)
Supplement: Supplementary file 1 [file plants-13-00187-s001.zip › Fig S1_Plants_Kim.pptx]

## Slide 1
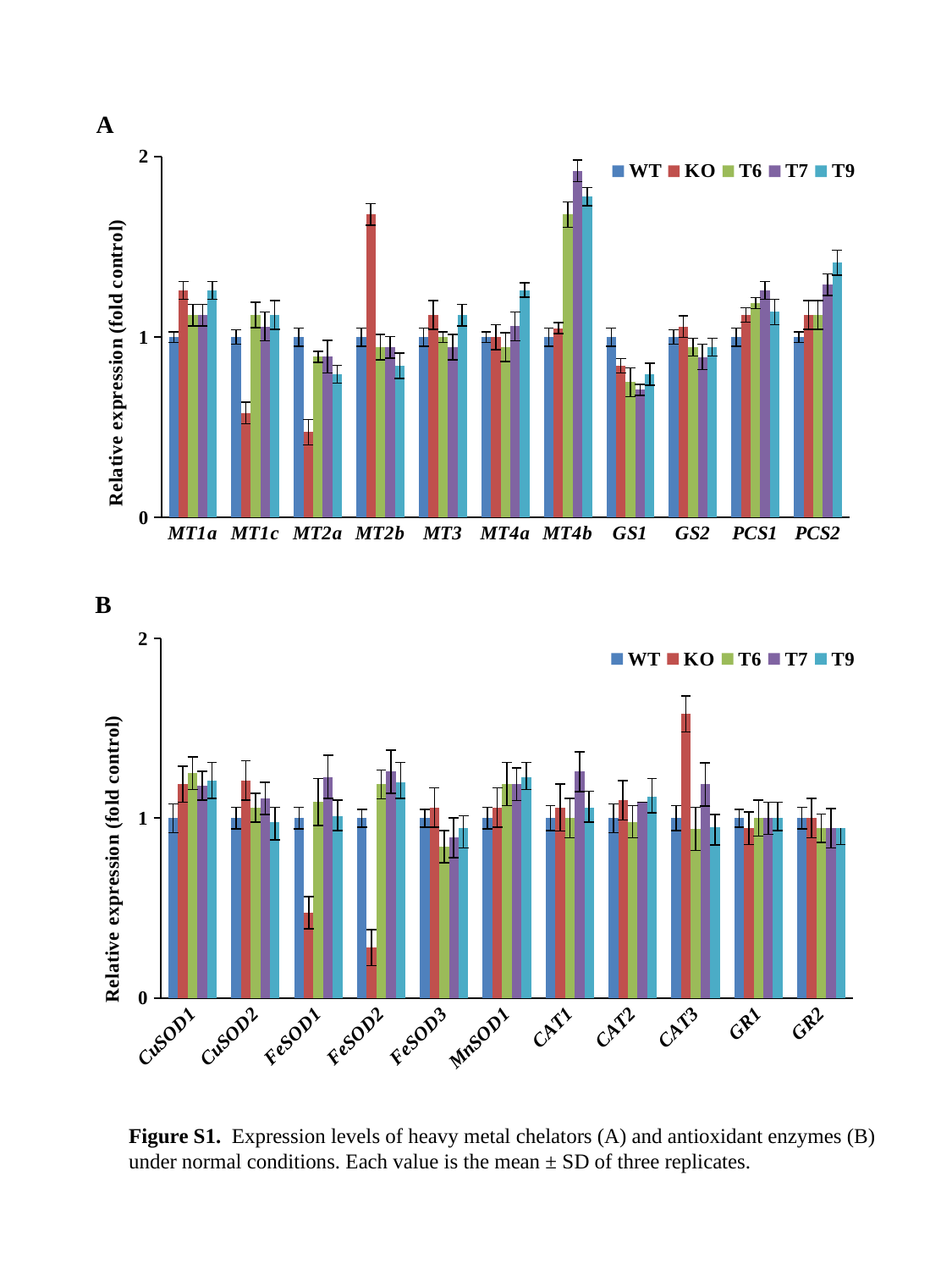

A
### Chart
| Category | WT | KO | T6 | T7 | T9 |
|---|---|---|---|---|---|
| MT1a | 1.0 | 1.2589021101824402 | 1.1220080704622584 | 1.1220080704622584 | 1.2589021101824402 |
| MT1c | 1.0 | 0.58 | 1.1220080704622584 | 1.059248823677542 | 1.1220080704622584 |
| MT2a | 1.0 | 0.4731797223371083 | 0.8912591863871424 | 0.8912591863871424 | 0.7943429373194711 |
| MT2b | 1.0 | 1.68 | 0.9440652447723855 | 0.9440652447723855 | 0.8414068219522148 |
| MT3 | 1.0 | 1.1220080704622584 | 1.0 | 0.9440652447723855 | 1.1220080704622584 |
| MT4a | 1.0 | 1.0 | 0.9440652447723836 | 1.06 | 1.26 |
| MT4b | 1.0 | 1.05 | 1.6787341042344108 | 1.92 | 1.778197125177668 |
| GS1 | 1.0 | 0.841406821952214 | 0.7499115595537207 | 0.707965440027724 | 0.7943429373194711 |
| GS2 | 1.0 | 1.059248823677544 | 0.9440652447723846 | 0.89 | 0.9440652447723846 |
| PCS1 | 1.0 | 1.1220080704622595 | 1.1884857287938582 | 1.2589021101824414 | 1.14 |
| PCS2 | 1.0 | 1.1220080704622606 | 1.1220080704622606 | 1.29 | 1.412498327546668 |B
### Chart
| Category | WT | KO | T6 | T7 | T9 |
|---|---|---|---|---|---|
| CuSOD1 | 1.0 | 1.19 | 1.25 | 1.18 | 1.21 |
| CuSOD2 | 1.0 | 1.21 | 1.0592488236775428 | 1.11 | 0.98 |
| FeSOD1 | 1.0 | 0.4731797223371073 | 1.09 | 1.23 | 1.01 |
| FeSOD2 | 1.0 | 0.28 | 1.1884857287938557 | 1.2589021101824402 | 1.2 |
| FeSOD3 | 1.0 | 1.059248823677544 | 0.8414068219522131 | 0.8912591863871406 | 0.9440652447723836 |
| MnSOD1 | 1.0 | 1.0592488236775428 | 1.19 | 1.1884857287938568 | 1.23 |
| CAT1 | 1.0 | 1.0592488236775428 | 1.0 | 1.2589021101824414 | 1.0592488236775428 |
| CAT2 | 1.0 | 1.1 | 0.98 | 1.09 | 1.12 |
| CAT3 | 1.0 | 1.58 | 0.94 | 1.1884857287938582 | 0.95 |
| GR1 | 1.0 | 0.9440652447723855 | 1.0 | 1.0 | 1.0 |
| GR2 | 1.0 | 1.0 | 0.9440652447723836 | 0.9440652447723836 | 0.9440652447723836 |Figure S1. Expression levels of heavy metal chelators (A) and antioxidant enzymes (B) under normal conditions. Each value is the mean ± SD of three replicates.
