# Supplementary material for: Glycine-Rich RNA-Binding Protein AtGRP7 Functions in Nickel and Lead Tolerance in Arabidopsis
Source: Plants (Basel). 2024 Jan 10;13(2):187. doi: 10.3390/plants13020187 (PMC10818801; doi:10.3390/plants13020187)
Supplement: Supplementary file 1 [file plants-13-00187-s001.zip › TabS1_Plants.pdf]

**Supplementary Table S1** Primers for qRT-PCR used in this study.

| | **Gene** | **Forward primer (5′ to 3′)** | **Reverse primer (5′ to 3′)** |
|---|---|---|---|
| Heavy metal Chelation | MT1a | CCTGCAAATGTGGTGACTCT | ACCCACAGCTGCAGTTTGAT |
| | MT1c | CCTGCAAATGTGGTGATTCGT | ACAGTTACAGCTTGACCCGCA |
| | MT2a | GCGAGACAACCACAACTGAG | TGCAGGTGCAAGGATCACAC |
| | MT2b | GTGGAAGCTGTGGTTGTGG | AACGAAAGTCTCGCCGGAAG |
| | MT3 | TGCTGACAAGACCCAGTGC | GCAAGTGCAGTTGACGCAG |
| | MT4a | ATCTGAATGTGGCAGAGGGAC | CATGTGCCCTTGATCCCCAG |
| | MT4b | TGCAGGTTAATGATGAGCG | CAAGTGCAACCCTTAGCAG |
| | GS1 | AGCATGTCGCTGAAGATGTC | AACACGGGATCTACGCTTTG |
| | GS2 | GTTCGTTTCCTGGCCTTAGTC | CCATGACTACCGCTCTTGGG |
| | PCS1 | TGGTGACTGGAGTTGTGGTG | CCCTGACCATGTCTGTGGAG |
| | PCS2 | TTGACCCGGGAAGAAAGTGG | TTTGTGCGGAAAGCTTCGAC |
| ROS Scavenging | CuSOD1 | GGTCCACATTTCAACCCCGA | CTGCATGGACAACAACAGCC |
| | CuSOD2 | GATGGCGTGGCAGAAACAAC | CCACCCTTTCCGAGGTCATC |
| | FeSOD1 | TCACCGCAAACTACGTCCTC | GAGCAGCGTTGTTGAAAGCA |
| | FeSOD2 | ACGGTTAGGGACAAAGGTGG | AATCCAAGGTTTCCCGGCTC |
| | FeSOD3 | GGATGTGTGGGAGCACTCTT | AGCGATTGGGATGTTGGGTT |
| | MnSOD1 | AATTCAACGGCGGAGGTCAT | GCACCCTCAGCACTCATCTT |
| | CAT1 | ACTACAGGCACATGGAAGGC | ATGGCTGTGATTGGCTCCTC |
| | CAT2 | GCTCGATGTGACCAAGACCT | GGCATAGGAGAAGACACGGG |
| | CAT3 | ACATGGAGGGTTTCGGTGTC | TTAGTGGCGTGGCTGTGATT |
| | GR1 | TCTGGACGCCAGGAAAAGAC | TGGATGTATCCCAACCGTGC |
| | GR2 | TGGGCTGTTGGGGATGTTAC | TCCAATAGGTGGCTGGGAGA |
| | Actin | CAGCAGAGCGGGAAATTGTAAGAG | TTCCTTTCAGGTGGTGCAACGAC |
